# New-Generation Antibiotics for Treatment of Gram-Positive Infections: A Review with Focus on Endocarditis and Osteomyelitis

**DOI:** 10.3390/jcm10081743

**Published:** 2021-04-17

**Authors:** Annemieke Bloem, Hannelore I. Bax, Erlangga Yusuf, Nelianne J. Verkaik

**Affiliations:** Department of Medical Microbiology and Infectious Diseases, Erasmus University Medical Center Rotterdam, 3015 GD Rotterdam, The Netherlands; h.bax@erasmusmc.nl (H.I.B.); e.yusuf@erasmusmc.nl (E.Y.); n.j.verkaik@erasmusmc.nl (N.J.V.)

**Keywords:** endocarditis, osteomyelitis, prosthetic joint infection, Gram-positive, new-generation antibiotics

## Abstract

Infective endocarditis, osteomyelitis, and osteosynthesis-associated infections are mostly caused by Gram-positive bacteria. They are often difficult to treat and are associated with a poor prognosis. In the past 20 years, nine antibiotic drugs with predominant activity against Gram-positive bacteria have been introduced and approved by the Food and Drug Administration or the European Medicines Agency: ceftaroline, daptomycin, telavancin, dalbavancin, oritavancin, linezolid, tedizolid, delafloxacin, and omadacycline. This narrative review aims to provide an overview on these antibiotics with a special focus on their use in infective endocarditis, osteomyelitis, and osteosynthesis-associated infections. Although some of these approved antibiotics are promising, they should not be used as first- or second-line therapy, awaiting more clinical data.

## 1. Introduction

Infective endocarditis (IE), osteomyelitis, and osteosynthesis-associated infections (OAI) are infectious diseases that are difficult to treat and associated with a poor prognosis [1,2]. The majority of these infections are caused by Gram-positive micro-organisms, including methicillin-resistant *Staphylococcus aureus* (MRSA) and vancomycin-resistant *Enterococcus faecium* (VRE). To treat these infections, usually a combination of surgery and antibiotic treatment is needed. However, antibiotic treatment may be challenging due to high bacterial loads, difficulty of antibiotics to achieve adequate drug concentrations at the site of infection, and biofilm formation [3,4].

In the past 20 years, several antibiotics with predominant activity against Gram-positive bacteria have been developed and approved by the Food and Drug Administration (FDA) or the European Medicines Agency (EMA). Although most of these antibiotics are not approved for these indications, they might also be used to treat IE, osteomyelitis, and osteosynthesis-associated infections. Previous reviews have been published on the use of just a few of these novel antibiotics in complicated infections [5,6]. Other reviews focused on the use of these novel antibiotics for approved indications [7,8,9,10,11]. Instead, the aim of this review is to provide an overview of nine of these new antibiotics with a special focus on their use in the treatment of IE, osteomyelitis, and osteosynthesis-associated infections. 

## 2. Materials and Methods

We first identified antibiotics with predominant activity against Gram-positive cocci that were approved by the FDA and/or EMA after 2000. Next, we performed a literature search in MEDLINE to identify (preferably clinical or, if not available, animal or in vitro) studies published or accepted for publication since 2000 on the use of these antibiotics for the indications as mentioned above. As such, the search term of each antibiotic agent was combined with “endocarditis”, “osteomyelitis”, “prosthetic joint infection”, and “bacteremia”. Studies were selected on the basis of title and abstract, only articles published in English were reviewed. Published studies on pediatric patients (<18 years) were not included.

## 3. Results

### 3.1. Ceftaroline

Ceftaroline is the only FDA and EMA approved beta-lactam antibiotic with activity against methicillin-resistant *Staphylococcus aureus* (MRSA), which is due to its high affinity for PBP2a [12]. Next to its in vitro activity against *S. aureus*, ceftaroline is active in vitro against coagulase-negative staphylococci (CoNS) and streptococci but shows no activity against *E. faecium*. The activity of ceftaroline against *Enterococcus faecalis* varies, with minimum inhibitory concentrations (MICs) between 0.12 and 32 mg/L [13,14].

Ceftaroline is approved by the FDA and EMA for acute bacterial skin and skin structure infection (ABSSSI) and community-acquired pneumonia (CAP) with an approved dose of 600 mg b.i.d. (bis in die/twice daily) IV (intravenous) (Table 1). Several studies showed that increasing the dose to 600 mg t.i.d. (ter in die/thrice daily), which can be used to treat infections due to *S. aureus* with ceftaroline MICs of ≥2 mg/L [15,16], is safe and well tolerated [17,18]. The clearance is predominantly renal and dose adjustment is required when creatinine clearance is below 50 mL/min (Table 1).

#### Ceftaroline in Infective Endocarditis and Osteomyelitis 

A phase IV registry (CAPTURE) included 55 patients with proven IE (26 right sided, 25 left sided, 4 bilateral) of which 80% was caused by MRSA [19]. Patients were treated for a median of 11 days (range 2–45 days). During this time, 41.8% of patients received ceftaroline monotherapy and 58.2% received ceftaroline with concurrent antibiotics (34.5% daptomycin, 16.4% vancomycin, and 12.7% rifampin). In all, 70.9% of the cases showed clinical cure or improvement (80.8% in right-sided IE, 68.0% in left-sided IE, but only 25% in bilateral IE). The monotherapy group improved in 82.6% of cases, whereas 62.5% of patients receiving combination therapy showed improvement. However, this could be due to the selection of patients. Of the 44 cases caused by MRSA, 77.3% achieved clinical success. No difference in success rate was observed between patients receiving 600 mg b.i.d. or t.i.d. 

The same registry evaluated 150 patients with osteomyelitis (62% caused by MRSA, 21.3% had osteosynthesis-associated infections) of whom 14.7% underwent surgery [20]. The majority (76%) of these patients received other antibiotics prior to ceftaroline (of which 54% received vancomycin). Moreover, 66.7% of patients (100/150) received ceftaroline monotherapy, and the rest of the patients received concurrent therapy (of which 36% were on metronidazole). Median treatment duration was 6 days (range 2–45 days). In this study, clinical success (i.e., cure or improvement based on individual physician assessment) was achieved in 92.7% of the patients. No data were presented on the subgroup with osteosynthesis-associated infections. 

For both indications, it is important to note the short duration of ceftaroline treatment in these studies. This could be partly explained by the fact that it was often used as second-line or rescue treatment. This makes it very complicated to draw conclusions on the efficacy of ceftaroline for these indications. The same applies for a retrospective analysis of 29 patients with MRSA bloodstream infections (BSIs), of whom 15 cases had IE (four right sided, 11 left sided), three cases had cardiac device infections, and nine cases had soft tissue or bone infection [21]. These patients received ceftaroline for a minimum of three days, and all patients received prior antibiotic treatment. No sub-analysis was performed for IE or osteomyelitis. Overall, in nine patients, treatment was considered successful, four patients were considered as treatment failures, and seven patients were lost to follow-up. 

Several studies have been published on the use of ceftaroline in bacteremia, whether or not in combination with other antimicrobial agents. Some of these studies also included IE and osteomyelitis cases.

In a retrospective study on 141 patients with BSI, the clinical success rate of ceftaroline therapy was 79% [22]. Part of these patients (approximately 30%) were treated with ceftaroline 600 mg t.i.d. Seventy-five patients with bone and joint infections were included, with an observed success rate in this group of 95%. No further information about this subgroup is provided.

A retrospective case control study on 32 patients with MRSA bacteremia showed higher microbiological and clinical success of ceftaroline compared to vancomycin, although not statistically significant [23]. A retrospective comparative study on MRSA BSI (among which there were seven IE and eight osteomyelitis cases) showed similar clinical success for ceftaroline monotherapy (n = 30) compared to daptomycin or vancomycin in terms of relapse, readmission, and 30 day mortality [24].

As for combination therapy with ceftaroline, evidence from in vitro studies indicates synergism between ceftaroline and daptomycin [25,26,27,28], which was retained in daptomycin-resistant isolates [28]. However, clinical studies are inconclusive. A multicenter observational study that included 211 patients with MRSA BSI showed a similar success rate for ceftaroline monotherapy compared to combination therapy (71.7% with daptomycin) [29]. A retrospective review described 11 patients with complicated MRSA BSI, treated with either ceftaroline and daptomycin or ceftaroline and vancomycin [30]. The microbiological cure was 100% for both treatment options, and there were no relapses at 30 or 60 days. A retrospective matched cohort study on MRSA BSI included 58 patients who were treated with daptomycin and ceftaroline and compared these with 113 patients who received standard of care (96% vancomycin) [31]. A lower mortality, a faster clearance of bacteremia and a lower rate of relapse and recurrence were observed with combination treatment, albeit not statistically significant. One open-label randomized controlled trial (RCT) in patients with MRSA BSI compared the duration of bacteremia with ceftaroline and daptomycin combination therapy to vancomycin or daptomycin monotherapy [32]. This study was terminated prematurely due to an unexpected observed in-hospital mortality difference of 0% (0/17) versus 26% (6/23) in favor of combination therapy with no difference in duration of bacteremia between the (small) groups. Larger prospective trials are needed in order to draw more solid conclusions on the role of ceftaroline–daptomycin combination therapy in this setting. 

### 3.2. Daptomycin

Daptomycin is a cyclic lipopeptide antibiotic with bactericidal activity against staphylococci, streptococci, and enterococci including VRE and MRSA [33]. It has different mechanisms of action including disruption of cell membrane function and inhibition of protein, DNA, and RNA synthesis.

Daptomycin is approved by the FDA and EMA for ABSSSI at a dose of 4 mg/kg/day IV and for *S. aureus* BSI and right-sided IE due to *S. aureus* at a dose of 6 mg/kg/day IV. Increasing the dose up to 10 mg/kg q.d. (quaque die/once daily) seemed to be tolerated well [34]. An important adverse event of daptomycin is elevation of creatine phosphokinase (CPK), which can be accompanied by rhabdomyolysis [35]. Therefore, at least once-weekly CPK monitoring is required. Clearance of daptomycin is predominantly renal, and dosage adjustment is required when creatinine clearance is <30 mL/min [33,36]. 

#### Daptomycin in Infective Endocarditis and Osteomyelitis

A study in predominately streptococcal native valve IE (NVIE) that compared 84 patients with a daptomycin-containing regimen (DCR, either monotherapy or in combination with other antibiotics) to 140 patients with non-daptomycin-containing regimens (non-DCR), showed that the 30 day mortality in patients with a DCR was higher than in the patients with a non-DCR (27.4% vs. 19.3%), albeit not significant [37]. In the same study, 61 patients with predominately staphylococcal prosthetic valve IE treated with daptomycin-containing regimens had significant lower 30 day mortality than those without daptomycin (6.5% vs. 38%, *p* < 0.001). This study also reported that the group of NVIE patients receiving a standard dose of daptomycin (4–6 mg/kg/day) showed a two times higher mortality risk (odds ratio 2.2, 95% CI 1.91–4.56, *p* = 0.02) compared to the group receiving a higher dose of daptomycin (>8 mg/kg/day). 

The efficacy of daptomycin seems to be highly dose dependent. One systematic review and meta-analysis on daptomycin use in VRE bacteremia showed a significantly higher 30 day mortality for daptomycin compared to linezolid [38]. However, in this study, no doses higher than 6 mg/kg/day were reported. Another systematic review and meta-analysis on this topic also showed a significantly higher 30 day mortality for daptomycin standard dose (6 mg/kg/day) compared to linezolid [39]. A subset analysis on high-dose daptomycin showed no difference in mortality compared to linezolid. Similarly, several publications on VRE and MRSA BSI and IE showed a survival benefit for daptomycin dosages of ≥7, 8, 9, or 10 mg/kg/day [40] compared to standard dose [41,42,43] or vancomycin [44].

The European Cubicin^®^ Outcomes Registry and Experience (EU-CORE) study investigated daptomycin treatment in 638 patients with osteomyelitis (224 cases of non-prosthetic osteomyelitis, 208 cases of osteomyelitis related to permanent or temporary orthopedic devices, and 206 cases with orthopedic device infections) [45,46]. A majority of the infections were caused by *S. aureus* (49.1% of the cases, of which half was MRSA), followed by CoNS (35.1%), streptococci, and enterococci. In this study, cure or improvement was shown in 81.8% of the patients (80.3% for osteomyelitis and 85% in prosthetic- or osteosynthesis-material-related infection). The majority (61.9%) of the patients also underwent surgical intervention and 71.3% received prior antibiotic treatment. 

A systematic review that summarized the outcome of 233 osteomyelitis patients (of which 184 were associated with osteosynthesis material and 115 with septic arthritis or osteomyelitis) treated with daptomycin confirmed the high clinical cure rate [46]. The clinical cure rate was 70% in patients with orthopedic device infections and 78% in osteomyelitis and septic arthritis cases. This study also showed that clinical cure rate (defined as total resolution of signs and symptoms or improvement to such an extent that no further antibiotic treatment was necessary) depends on the dose. Clinical cure rate was 85% in patients who received a dose of 10 mg/kg/day (interquartile range (IQR) 67–92), 85% for 6–8 mg/kg/day (IQR 63–95), and 71% for 4–6 mg/kg/day (IQR 58–80).

As for daptomycin combination therapy, several studies have been published. For daptomycin in combination with rifampin, in vitro models show diverse results from possible antagonism in an experimental endocarditis model [47] to increased efficacy [48] and bactericidal activity in biofilm-embedded bacteria in *S. aureus* biofilms [49]. Increased efficacy was also shown for combination therapy in an experimental MRSA foreign-body-infection model [50]. In response to the experimental endocarditis study [47] with possible antagonism, in vitro checkerboard assays were performed, which did not show antagonism [51].

In an experimental endocarditis model in rabbits, daptomycin and fosfomycin showed synergy and rapid bactericidal activity [52]. In an RCT on MRSA BSI and IE with 155 patients, treatment success rate was 12% higher for daptomycin in combination with fosfomycin compared to daptomycin monotherapy, albeit not statistically significant [53]. 

As discussed in the ceftaroline section, daptomycin and ceftaroline combination therapy might have a positive effect on clinical outcome [32].

### 3.3. Telavancin

Telavancin is a semisynthetic lipoglycopeptide antibiotic engineered to be an improved alternative to the available glycopeptides, vancomycin and teicoplanin. It shows in vitro activity against methicillin-sensitive *S. aureus* (MSSA), MRSA, CoNS, streptococci, and VRE *vanB* but not *vanA* [54].

Telavancin is FDA approved for ABSSSI, hospital acquired pneumonia (HAP), and ventilator-assisted pneumonia (VAP) in a dosing regimen of 10 mg/kg/day. It was initially EMA approved for use in nosocomial pneumonia and VAP caused by MRSA but was withdrawn in 2018 because of commercial reasons [55]. Clearance is predominantly renal, and dose adjustment is needed when creatinine clearance is <50 mL/min [56]. Telavancin is associated with increased creatinine levels more often than vancomycin [57]. Moreover, adverse drug reactions led to the discontinuation of therapy more often in the telavancin-treated patients (8%) than in the vancomycin-treated patients (5%).

The FDA required a Risk Evaluation and Mitigation Strategy (REMS), as part of the drug safety program, because an increased mortality rate was observed in telavancin-treated patients with HAP/VAP with a pre-existing creatinine clearance of ≤50 mL/min, and also fetal toxicity was observed in animal models [58,59]. Since 2017, a REMS is no longer required [60].

#### Telavancin in Infective Endocarditis and Osteomyelitis

Published data on the use of telavancin in IE is limited and mostly concerns case reports [61,62,63]. One case series that included 14 cases treated with telavancin for refractory MRSA bacteremia after antibiotic treatment with vancomycin or daptomycin showed that 8 of 14 patients (57%) survived inpatient admission and were eligible for follow-up [64]. Four of them underwent surgery. Patients who died all had mitral valve IE. Among the six patients who died, one underwent valve replacement and the other five cases were not considered surgical candidates. The median duration of bacteremia was 12 days (range 3–26 days). Patients received vancomycin and, in some cases, daptomycin. Median time to achieve sterilization (in 10 patients) was 1 day (1–3 days) following initiation of telavancin.

In a large observational study, 151 patients with bacteremia (13 with IE) were treated with telavancin (57.6% MRSA) [65]. The overall median treatment duration was 9 days but was not specified for IE. Among the 11 IE patients for whom follow-up data were available, eight patients had a positive outcome (seven were cured and one improved; three patients failed) at end of telavancin therapy [65]. In this study, overall, 74.2% had a positive outcome, defined as either cure or partial response (with possible further need for telavancin therapy). 

Three observational studies have been published regarding telavancin for the treatment of osteomyelitis and osteosynthesis-associated infections. These studies included 14 [66], 60 [67], and 32 [68] patients with osteomyelitis, of which 64%, 37%, and 15% had osteosynthesis material, respectively. In the first study, surgery was performed in 57% of patients (8/14), but this was not specified in the latter two. Clinical success, defined as resolved or improved infection, was 78%, 73%, and 92% with a median treatment duration of 58 days (9–66), 41 days (3–179), and 31.5 days (6–94), respectively. MRSA was the most frequently isolated pathogen (100%, 67%, and 57%, respectively) [66,67,68].

For uncomplicated MRSA bacteremia, telavancin has been proven to be equally or more effective compared to vancomycin [69,70]. A large observational study described 1065 patients who were treated with telavancin for a median treatment duration of 10 days (range 5 – 26 days) [71]. Positive clinical outcome, either cure (defined as resolution of signs and symptoms and no need for additional therapy) or improvement to stepdown therapy, was reached in 77.7%. Of these 1065 cases, 27.4% concerned bone and joint infections and 14.2% IE, a positive outcome was reported in 74.2% and 78.7% of the cases, respectively. No further information on these subgroups was reported. 

### 3.4. Dalbavancin

Dalbavancin is a semisynthetic lipoglycopeptide antibiotic, with a half-life of approximately 8.5 days, enabling weekly dosing regimens or single-dose therapy in the treatment of ABSSSI. It shows in vitro activity against MSSA, MRSA, CoNS, and streptococci, *E. faecalis* and *E. faecium,* including VRE *vanB* but not *vanA* [72].

Dalbavancin is approved by the FDA and EMA for ABSSSI in a dosing regimen of 1500 mg IV as a single dose or alternatively 1000 mg IV on day 1, followed by 500 mg IV on day 8. Both regiments have similar rates of adverse events [73,74]. Dalbavancin is predominately excreted via the urine. Dosage adjustment is recommended when renal clearance is <30 mL/min [72].

#### Dalbavancin in Infective Endocarditis and Osteomyelitis

A retrospective multicenter study on off-label use of dalbavancin for IE (n = 25), osteomyelitis (n = 30), OAI (n = 32), ABSSSI, and catheter-related BSI showed promising results, with an 89% overall success rate (defined as no clinical, microbiological, or laboratory evidence of persistent or recurring infection at 90 days), with particular good results for IE (91.3%) and OAI (92.7%) [75]. Different dosing regimens were used, and the median number of administrations was three. No information was provided on whether or not surgery is performed.

A clinical cure rate (i.e., no BSI recurrence or IE relapse/reinfection, no mortality) of 85.3% was observed during a 12 month follow-up in an observational study assessing dalbavancin efficacy in 83 patients with BSI, of whom 34 had proven IE (20.6% MSSA, 8.8% MRSA, 44.1% coagulase-negative staphylococci (CoNS), 20.6% streptococci, and 8.8% *E. faecalis*) [76]. All patients received prior antibiotics, and surgery was performed in 10 of 15 patients with prosthetic valve IE, 7 of 8 pacemaker lead IE, and 5 out of 11 native valve IE. 

A high proportion of clinical cure (92.6%) was also observed in an observational study of 27 patients with proven IE, of which there were 16 native valve, 6 prosthetic valve, and 5 cardiac device-related IE, caused by CoNS (76.7%) and *S. aureus* (33.3%) [77]. Patients were treated with dalbavancin with a median treatment duration of six weeks (range 1–30 weeks) and combined with surgery in 11 patients. In this study, failures were observed in two patients only (one due to non-infective postoperative complications and one due to incomplete surgical source control). However, the majority of the patients (24 of 27) received other antibiotics and already had microbiological clearance prior to dalbavancin; so, it was not clear whether the clinical success was attributable to dalbavancin. The three patients who received dalbavancin as primary therapy showed no relapse at six months follow-up. 

One RCT has been published on dalbavancin treatment for osteomyelitis. In this RCT, which did not include patients with osteosynthesis-associated infections, patients were randomized to a two-dose regimen of dalbavancin 1500 mg on day 1 and 8 (n = 70), or standard-of-care (n = 10, n = 3 vancomycin monotherapy, n = 7 vancomycin combined with either linezolid or levofloxacin) [78]. All patients underwent baseline debridement. In this study, MSSA and MRSA were causative pathogens in 54.3% and 5.7% of cases, respectively. The primary endpoint, defined as recovery without the need for additional antibiotic treatment at day 42, was reached in 97% in the dalbavancin group and 88% in the comparator group. Clinical significance was not reported, probably due to the small comparator group. 

In an observational study with prolonged use of dalbavancin (median five doses), 64 patients with osteomyelitis and septic arthritis (45 with osteosynthesis-associated infections) were included [79]. In the patients with osteosynthesis material, *S. epidermidis* was the most common causative pathogen, in the rest of the patients the most common causative pathogen was *S. aureus* (93.9% MRSA). Clinical cure (i.e., absence of clinical signs of infection at the latest medical visit, without the need for additional surgery or antibiotic treatment) was reached in 68.8% of patients with osteosynthesis-associated infections (65.2% with implant retention and 76.1% with implant removal) and 73.6% in the remaining patients. 

For catheter-related BSI, in a phase 2 study with 75 patients, dalbavancin therapy showed a significantly higher success rate than vancomycin (87% vs. 50%) [80]. Furthermore, dalbavancin seems to be a promising option for outpatient parenteral antibiotic therapy [81] or long-term suppression therapy [82].

As for dalbavancin combination therapy, an MRSA foreign-body-infection model with infected cages in guinea pigs was used to determine the efficacy of dalbavancin in combination with rifampin [83]. Dalbavancin in combination with rifampin cured 36% of the infected cages. A similar cure rate was observed with rifampin monotherapy. During rifampin monotherapy, rifampin resistance was observed in 38% of the treatment failures. In combination therapy, rifampin resistance was reduced from 25% to 0% by increasing dalbavancin dosages from 40 up to 80 mg/kg. No dalbavancin resistance was observed. 

### 3.5. Oritavancin

Oritavancin is another semisynthetic lipoglycopeptide antibiotic with a long half-life, providing the opportunity of weekly dosing. The spectrum of activity is largely similar to that of dalbavancin but with additional activity against VRE *vanA* [84,85]. 

Oritavancin is approved by the FDA and EMA for ABSSSI as a single dose of 1200 mg IV. Its clearance is predominantly renal, but dose adjustment is not recommended in mild-to-moderate renal impairment. No pharmacokinetic data are available for severe renal impairment [84,86]. In contrast to dalbavancin, oritavancin is a weak inhibitor of certain cytochrome P450 enzymes [84,87,88]. Oritavancin also interacts with several laboratory tests due to its ability to bind to the phospholipid reagent, preventing the activation of coagulation in commonly used laboratory coagulation tests, among which were activated Partial Thromboplastin Time (aPTT) and Prothrombin Time (PT) [89].

#### Oritavancin in Infective Endocarditis and Osteomyelitis

The long half-life of oritavancin provides clear practical advantages in the treatment of infections that need long-term antibiotic treatment such as IE and osteomyelitis. Weekly dosing can improve compliance. However, only a limited number of papers (case reports) have been published on the use of oritavancin, especially for IE.

In a case report, a patient with recurrent VRE bacteremia due to native aortic and mitral valve IE was successfully treated with 10 weeks of oritavancin treatment (1200 mg, twice weekly) in combination with valve replacement surgery [90].

A recent, multicenter, retrospective descriptive study described 134 patients with acute osteomyelitis [91]. They received four or five doses of oritavancin (1200 mg, followed by 800 mg weekly). MRSA was the cause for 71.9%, while 6.7% had concurrent MRSA BSI. Clinical success (defined as resolution of symptoms or improvement of symptoms and no further need for treatment) was achieved in 88.1%. Debridement was performed in 90.3% of the cases. Another retrospective observational program described 438 patients who received at least one dose of oritavancin [92]. Among them, 32 cases were considered complicated infections, of which 18 cases concerned osteomyelitis, three concerned osteosynthesis-associated infections, and seven patients had BSI. Clinical success (defined as cure or improvement after 30 days) was achieved in 93.8% of the complicated infections. For the patients who received one single dose, clinical success was 87.7% and for multiple dosages it was 93.7%. Prior antibiotics were administered in 71.4% of the cases. 

### 3.6. Linezolid

Linezolid is a synthetic bacteriostatic agent of the oxazolidinone class of antibiotics and selectively inhibits bacterial protein synthesis. Linezolid is the only antibiotic drug mentioned in this review that is approved for the treatment of VRE infections, including bacteremia, and is available both in IV and PO formulation. Linezolid shows in vitro activity against MSSA, MRSA, CoNS, streptococci, and enterococci, including *vanA* and *vanB* VRE [93].

Linezolid is approved by the FDA and EMA for ABSSSI, HAP, and CAP. The FDA also approved linezolid for infections due to VRE, including VRE bacteremia. Its approved dosing regimen is 600 mg b.i.d. IV/PO. Linezolid has an oral bioavailability of 100% with or without food [94,95]. Linezolid is predominantly excreted (partly unchanged) in urine. No dose adjustment is needed in renal failure [93,96]. When the treatment duration is shorter than two weeks, linezolid is generally well tolerated, but longer treatment duration is associated with an increased risk of myelosuppression, in particular reversible thrombocytopenia [97,98]. Therefore, patients with (prolonged) linezolid treatment should be monitored for myelosuppression, particularly in patients with renal impairment [99]. 

#### Linezolid in Infective Endocarditis and Osteomyelitis

In a systematic review of published case reports, cure (defined as clinical improvement, negative blood cultures, and no evidence of persistent vegetation on echocardiography) was investigated in 33 IE patients. Of the patients, 66.7% received linezolid monotherapy, 84.8% received prior antibiotic treatment, and 24% underwent surgical intervention [100]. MRSA and vancomycin-intermediate *S. aureus* were the most commonly isolated cocci (24.2% and 30.3% of cases, respectively). Cure was shown in 63.6% of the patients. Higher success rates were reported in two small observational studies. In a study of 14 patients, in which linezolid was used as follow-up therapy for 3 weeks and all patients underwent valve replacement surgery and prior vancomycin treatment, a 100% success rate (i.e., clearance of symptoms and the absence of persistent bacteremia) was found [101]. Another study that included nine patients (<50% underwent surgery, 44.4% was caused by MSSA, and 22.2% by MRSA and all received prior antibiotics for a median length of 22 days) showed a clinical cure rate of 100%, with no relapses during follow-up (mean follow-up duration of 8.5 months) [102].

A study that assessed linezolid efficacy in 55 patients with osteomyelitis (70.9% without prosthetic material, 16.4% with prosthetic material removed, and 12.7% without prosthetic material removal), showed clinical cure, defined as the resolution of signs and symptoms, in 81.8% after a median of 189 days [103]. Subgroup analysis in patients with osteomyelitis caused by VRE showed a 93.9% cure rate at short-term follow-up (7 to 10 days after discontinuation of linezolid). One patient with VRE infection was available for long-term follow-up (one month after discontinuation of linezolid), and this patient was cured. Smaller studies also show high cure rates. As such, a 100% cure rate (i.e., clinical recovery, normalization of inflammatory markers, and no need for reoperation or rehospitalization) was shown in a study in 22 patients with chronic osteosynthesis-associated infection (18 associated with fracture fixation and four with arthroplasties), with 14 patients available for follow-up after 6 months [104]. All patients underwent surgery (debridement and removal of all implants), and 45% of the infections were caused by MRSA. Use of antibiotics other than linezolid was not specified. Another study in 11 patients with osteomyelitis (45% of the infections were caused by MRSA, all patients underwent surgery), of which two were implant-related, also showed 100% remission according to clinical, laboratory, and radiographic criteria [105].

Linezolid is approved by the FDA for the treatment of VRE BSI. Comparative studies of linezolid and daptomycin are discussed in the daptomycin section.

In the case of persistent MRSA bacteremia (≥7 days), linezolid proved to be statistically more effective than vancomycin in terms of early microbiological response, success rate, and mortality rate [106]. However, statistical significance for better outcome was not confirmed by another study on the same topic [107].

As for linezolid combination therapy, an in vitro model for staphylococcal biofilm showed significantly increased biofilm activity of linezolid in combination with rifampin compared to that of linezolid monotherapy [108]. This combination did not lead to rifampin resistance. However, levofloxacin with or without rifampin was still more effective. A retrospective study compared linezolid in combination with rifampin to cotrimoxazole in combination with rifampin in 56 bone and joint infections [109]. No difference was observed in either efficacy or tolerance. 

### 3.7. Tedizolid

Like linezolid, tedizolid belongs to the oxazolidinone class of drugs and is available in an oral formulation with the same spectrum of activity, including activity against VRE [110,111].

Tedizolid is approved for ABSSSI by the FDA and EMA in a dosing regimen of 200 mg q.d. PO or IV. Oral bioavailability is 91% and independent of food intake [112]. Clearance is predominantly hepatic, but dose adjustment is not required in both hepatic and renal impairment [113]. Treatment duration of 6 days for ABSSI was shown to be non-inferior to 10 days of linezolid therapy [114,115]. Like linezolid, tedizolid is associated with thrombocytopenia, albeit with lower frequency than linezolid [116], which might be partly explained by the shorter treatment duration of tedizolid (6 days) compared to that of linezolid (10 days). Similar to linezolid, the proportion of patients with thrombocytopenia has been shown to increase when the treatment duration is prolonged and in patients with chronic renal failure.

#### Tedizolid in Infective Endocarditis and Osteomyelitis

To the best of our knowledge, no clinical data on tedizolid efficacy in patients with IE and osteomyelitis have been published. An in vitro study in a biofilm model showed that tedizolid was not effective in eradicating mature *S. aureus* and *S. epidermidis* biofilms [117].

A rabbit model of MRSA aortic valve IE showed that tedizolid and vancomycin were less effective than daptomycin after 4 days of treatment [118]. However, in a rat endocarditis model, the efficacy of 2 days of tedizolid step-down therapy after 3 days of daptomycin administration was similar to that of daptomycin for 5 days, which might suggest a role for tedizolid step-down therapy [119]. Caution is needed in extrapolating these results to humans. 

In a rat model of MRSA and methicillin resistant *Staphylococcus epidermidis* (MRSE) osteosynthesis-associated infections, tedizolid for 21 days, either alone or in combination with rifampin, reduced bacterial counts [120,121]. This was comparable to vancomycin with or without rifampin, and bacterial counts were significantly lower than in the non-treatment group. 

As for combination therapy, in an in vitro model of endocardial vegetations, antagonism was observed when tedizolid was combined with daptomycin [122].

### 3.8. Delafloxacin

Delafloxacin belongs to the fluoroquinolone class of drugs and is active in vitro against MSSA, MRSA, CoNS, and streptococci. Interestingly, delafloxacin retains activity against fluoroquinolone-resistant *S. aureus* strains [123,124,125,126]. Specific features in the delafloxacin molecule lead to enhanced activity in acidic environment due to its anionic character, which eventually leads to improved activity [127,128]. According to EUCAST, there is insufficient evidence that enterococci are a good target for therapy with delafloxacin. 

Delafloxacin was approved by the FDA and EMA for ABSSSI and additionally for CAP by the FDA. The approved dose is 300 mg b.i.d. IV and 450 mg b.i.d. PO. Oral bioavailability is approximately 58–70% [129,130]. The oral dose of 450 mg leads to a similar total exposure to that of 300 mg IV and is not affected by food intake [129,131]. Clearance is predominantly renal, and dose adjustment for the IV formulation is required when creatinine clearance is below 30 mL/min. No adjustment is needed either for the oral formulation or in hepatic impairment [132]. Like other fluoroquinolones, delafloxacin may be associated with increased risk of serious adverse events, such as tendon ruptures and tendinitis, and aneurysmatic and neurological complications, for which the FDA issued a fluoroquinolone box warning [133]. Since specific data on delafloxacin are still limited, the occurrence of side effects should be monitored closely [134].

#### Delafloxacin in Infective Endocarditis and Osteomyelitis

No clinical data have been published on the use of delafloxacin for IE and osteomyelitis. In general, other fluoroquinolones such as ciprofloxacin, levofloxacin, and moxifloxacin are known to be efficacious in treating osteomyelitis, including osteosynthesis-associated infections, due to their ability to eradicate biofilm formation on osteosynthesis material surface [123]. An in vitro study using biofilm models with 96-wells plates confirms the ability of delafloxacin to reduce *S. aureus* biofilm viability and depth [135,136]. In this study, its biofilm penetration was also deeper in comparison to that of daptomycin and vancomycin, perhaps due to its smaller molecular mass and increased activity in an acidic environment [127,136].

### 3.9. Omadacycline

Omadacycline is a member of a new subclass of tetracyclines. It shows in vitro activity against MSSA, MRSA CoNS, streptococci, and enterococci including VRE [137,138]. 

Omadacycline is FDA approved for the treatment of ABSSSI and CAP and is available both in IV and oral formulation. The approved dosing regimen for ABSSSI requires a loading dose of 200 mg q.d. IV or 100 mg b.i.d. IV on day 1, or 450 mg q.d. PO on day 1 and 2. The maintenance dose is 100 mg q.d. IV or 300 mg q.d. PO. Despite limited oral bioavailability (34.5%), similar exposure can be achieved with 300 mg PO as with 100 mg IV [139]. Adequate oral bioavailability can only be achieved in the fasted state ≥4 h before and 2 h after intake. EMA approval of omadacycline was withdrawn in 2019 by the authorization holder because, according to EMA, the benefits of the drug did not outweigh the risks. Although omadacycline treatment in patients with CAP was non-inferior compared to moxifloxacin treatment, an unexplained imbalance in mortality rate was noticed at the expense of omadacycline [140]. Although this could not be solely attributed to omadacycline, it did lead to a negative benefit-to-risk ratio according to the EMA [141]. Approval for ABSSSI only was not considered advantageous from a commercial point of view. Omadacycline is primarily excreted unchanged in feces and secondarily excreted in urine [139], but no dose adjustment is needed in impaired renal or hepatic function [142]. Omadacycline has comparable adverse events to other tetracyclines such as tooth discoloration, enamel hypoplasia, and inhibition of bone growth. However, no cases of photosensitivity were observed in the phase III registration studies that included 1073 patients [143].

#### Omadacycline in Infective Endocarditis and Osteomyelitis

No clinical data or case reports are available on omadacycline use in IE or OSM/prosthetic joint infection (PJI). Omadacycline distributes extensively throughout the body, and excellent bone tissue penetration has been shown in animal models [138,139].

## 4. Discussion and Concluding Remarks

We reviewed nine antibiotics with predominant Gram-positive activity and focused on the treatment of IE and osteomyelitis (including osteosynthesis-associated infections). For some antibiotics such as ceftaroline, daptomycin, and linezolid, a number of clinical studies are available. For most of the described antibiotics, however, clinical data are limited. It is difficult to assess the efficacy because the evidence is mostly observational, and patients often received prior or concurrent antibiotics. Dosing regimens are quite diverse, and in some studies patients, were included after receiving only one dose of the study drug. Furthermore, for IE and OAI, surgical intervention is frequently necessary, and whether or not this is performed might have significantly impacted treatment results. 

Another limitation of this narrative review is the risk of bias, particularly selection bias, with incomplete retrieval of publications as a result. However, the main goal of this narrative review was to assess the most important data on the efficacy of these novel antibiotics in order to provide some guidance on their application in clinical practice. As such, this review is considered appropriate for this purpose. 

For most antibiotics, studies have been published about combination therapy in in vitro models or animal studies. Although results might seem promising, in most cases, results have not been confirmed in human studies yet. Caution is needed in extrapolating these findings to the clinical setting, even more because of possible antagonism as might have been observed in the preclinical setting.

In order to use these new-generation antibiotics, antimicrobial susceptibility tests and breakpoints should be available in the microbiology laboratories. For all antibiotics reviewed here, except for omadacycline, EUCAST breakpoints for staphylococci are available (EUCAST version 10.0). For streptococci, EUCAST breakpoints are available for all agents except for telavancin and omadacycline. For enterococci, only linezolid EUCAST breakpoints are available. Clinical and Laboratory Standards Institute breakpoints may be used to overcome this problem.

In conclusion, promising antibiotics for the treatment of Gram-positive infections have been approved in the last 20 years. However, these new antibiotics should not be used as first-line therapy as clinical data are limited. Only in case of resistance to first- and second-line agents, allergy, or in clinical and/or microbiological failure should these antibiotics be considered. In these cases, the practical approach in this review, including the summary table, could be helpful to determine which of these antibiotics would be the preferable agent to use.

## Figures and Tables

**Table 1:** General features of nine Food and Drug Administration (FDA)- or European Medicines Agency (EMA)- approved antibiotic drugs with Gram-positive activity discussed in this paper.

	Cephalosporins	Lipopeptides	Lipoglycopeptides			Oxazolidinones		Fluoroquinolones	Tetracyclines
	Ceftaroline	Daptomycin	Telavancin	Dalbavancin	Oritavancin	Linezolid	Tedizolid	Delafloxacin	Omadacycline
In vitro activity	MSSA, MRSA, CoNS, streptococci, some *Enterococcus faecalis* isolates	MSSA, MRSA, CoNS, streptococci, enterococci including VRE *vanA, vanB*	MSSA, MRSA, CoNS, streptococci, enterococci including VRE *vanB*	MSSA, MRSA, CoNS, streptococci, enterococci including VRE *vanB*	MSSA, MRSA, CoNS, streptococci, enterococci including VRE *vanA, vanB*	MSSA, MRSA, CoNS, streptococci, enterococci including VRE *vanA, vanB*	MSSA, MRSA, CoNS, streptococci, enterococci including VRE *vanA, vanB*	MSSA, MRSA, CoNS, streptococci, *E. faecalis*	MSSA, MRSA, CoNS, streptococci, enterococci including VRE *vanA, vanB*
No activity	*Enterococcus faecium,* VRE *vanA, vanB*		VRE *vanA*	VRE *vanA*				*E. faecium,* VRE *vanA, vanB*	
Drug target	Cell wall synthesis	Cell wall synthesis	Cell wall synthesis	Cell wall synthesis	Cell wall synthesis	Protein synthesis	Protein synthesis	DNA replication	Protein synthesis
FDA/EMA approved dosing regimen (for ABSSSI, unless otherwise mentioned)	600 mg b.i.d. IV	ABSSSI 4 mg/kg/day IVBSI/IE 6 mg/kg/day IV	10 mg/kg/day IV	1500 mg IV single doseAlternative: 1000 mg IV single dose at day 1, followed by 500 mg IV single dose at day 8	1200 mg IV single dose	600 mg b.i.d. IV / PO	200 mg q.d. IV / PO	300 mg b.i.d. IV OR450 mg b.i.d. PO	Loading dose: - IV: 200 mg q.d. on day 1 OR 100 mg b.i.d on day 1- PO (for ABSSSI only): 450 mg q.d. on day 1 and 2. Maintenance: - IV: 100 mg q.d. OR- PO: 300 mg q.d.
Recommended dosing regimen for IE and OSM/PJI	600 mg b.i.d. - t.i.d. IV (15-18)	6-12 mg/kg/day IV (34, 37, 46)	No data	No data	No data	No data	No data	No data	No data
Dose adjustments	Creatinine clearance: - 30-50 mL/min: 400 mg b.i.d. IV; - 15-30 mL/min 300 mg b.i.d.; - ESRD/HD: 200 mg b.i.d.	Creatinine clearance <30 mL/min, HD, CAPD: 6 mg/kg IV every 48 h	Creatinine clearance:- 30-50 mL/min 7.5 mg/kg/day - < 30 mL/min 10 mg/kg every 48 h	Creatinine clearance < 30 mL/min: 750 mg IV single dose at day 1, followed by 375 mg IV at day 8	Not recommended in mild or moderate impairment, pharmacokinetics in patients with severe renal impairment has not been evaluated	None	None	Creatinine clearance 15-29 mL/min: 200 mg b.i.d. IV. No dose adjustment PO.	None
Points of interest	Only approved cephalosporin active against MRSA	Not indicated for pneumoniaMonitor CPK and renal function once weekly	Renal dysfunction observed more often compared with vancomycin	Single dose therapy	Single dose therapy	Oral formulation available with 100% oral bioavailabilityRisk of myelosuppression after prolonged use >14 days, monitoring recommended	Oral formulation available with 91% oral bioavailability	Oral formulation available with 58.8% oral bioavailability	Oral formulation availableAdequate oral bioavailability only in fasted state (≥4 h before and 2 h after dose)
FDA approval (year, brand name, and indications)	2010, Teflaro^®^, ABSSSI, CAP	2003, Cubicin^®^, ABSSSI, right-sided infective endocarditis SA, bacteremia SA	2009, Vibativ^®^, ABSSSI, HAP, VAP	2014, Xydalba^®^, ABSSSI	2014, Orbactiv^®^, ABSSSI	2000, Zyvox^®^, - ABSSSI (including diabetic foot infection, without osteomyelitis) caused by MSSA, MRSA, *Streptococcus pyogenes, Streptococcus agalactiae*= HAP caused by MSSA, MRSA, *Streptococcus pneumoniae*- CAP caused by *S. pneumoniae* (incl. BSI), MSSA- VRE infections incl. BSI	2014, Sivextro^®^, ABSSSI	2017 and 2019, Baxdela^®^ ABSSSI (2017), CAP (2019)	2018, Nuzyra^®^, ABSSSI, CAP
EMA approval (year, brand name, and indications)	2012, Zinforo^®^, ABSSSI, CAP	2006, Cubicin^®^, ABSSI, right-sided infective endocarditis SA, bacteremia SA	Not EMA approved	2015, Xydalba^®^, ABSSSI	2015, Orbactiv^®^, ABSSSI	2001, Zyvox^®^, ABSSSI, HAP, CAP	2015, Sivextro^®^, ABSSSI	2019, Quofenix^®^, ABSSSI	Not EMA approved

Abbreviations: MSSA, methicillin-sensitive *Staphylococcus aureus;* MRSA, methicillin-resistant *S. aureus;* CoNS, coagulase-negative staphylococci; VRE, vancomycin-resistant *E. faecium;* ABSSSI, acute bacterial skin and skin structure infections; BSI, bloodstream infections; IE, infective endocarditis; CAP, community-acquired pneumonia; HAP, hospital-acquired pneumonia; VAP, ventilator-assisted pneumonia. IV, intravenous; PO, per os; OSM; osteomyelitis; PJI, prosthetic joint infection; q.d., quaque die/once daily; b.i.d., bis in die/twice daily; t.i.d., ter in die/three times daily; CAPD, continuous ambulatory peritoneal dialysis; ESRD, end-stage renal disease; HD, hemodialysis; CPK, creatinine phosphokinase; FDA, Food and Drug Administration; EMA, European Medicines Agency; SA, *S. aureus.*
